# The pons as an optimal background reference region for spinal ^18^F-FET PET/MRI evaluation

**DOI:** 10.1186/s13550-024-01130-5

**Published:** 2024-07-26

**Authors:** Jing Huang, Jiyuan Wang, Bixiao Cui, Hongwei Yang, Defeng Tian, Jie Ma, Wanru Duan, Zan Chen, Jie Lu

**Affiliations:** 1 Department of Radiology and Nuclear Medicine, Xuanwu Hospital, Capital Medical University, 45 Changchun Street, Xicheng District, Beijing, China; 2 Beijing Key Laboratory of Magnetic Resonance Imaging and Brain Informatics, Capital Medical University, Beijing, China; 3 Department of Neurosurgery, Xuanwu Hospital, Capital Medical University, Beijing, China

**Keywords:** Hybrid PET/MR, Standard uptake value ratio, Background reference region, Spinal cord tumor, Myelitis

## Abstract

**Background:**

This study aims to evaluate the effect of various background reference regions on spinal ^18^F-FET PET imaging, with a focus on distinguishing between spinal tumors and myelitis. To enhance diagnostic accuracy, we investigated the pons and several other spinal cord area as potential references, given the challenges in interpreting spinal PET results.

**Results:**

A retrospective analysis was conducted on 30 patients, 15 with cervical myelitis and 15 with cervical tumors, who underwent O-(2-[^18^F]-fluoroethyl)-L-tyrosine (FET) PET/MR imaging. The stability of uptake across four regions, including the pons, C2, C2–C7, and T1–T3, was compared. The standardized uptake value ratio (SUVR) was then evaluated using various background regions, and their effectiveness in differentiating between spinal tumors and myelitis was compared. Additionally, we correlated the SUVR values derived from these regions with the Ki-67 proliferation index in tumor patients. The study found no significant difference in SUVmax (U = 110, *p* = 0.93) and SUVmean (U = 89, *p* = 0.35) values at lesion sites between myelitis and tumor patients. The pons had the highest average uptake (*p* < 0.001) compared to the other three regions. However, its coefficient of variation (CV) was significantly lower than that of the C2–C7 (*p* < 0.0001) and T1–T3 segments (*p* < 0.05). The SUVRmax values, calculated using the regions of pons, C2–C7 and T1–T3, were found to significantly differentiate between tumors and myelitis (*p* < 0.05). However, only the pons-based SUVRmean was able to significantly distinguish between the two groups (*p* < 0.05). Additionally, the pons-based SUVRmax (*r* = 0.63, *p* = 0.013) and SUVRmean (*r* = 0.67, *p* = 0.007) demonstrated a significant positive correlation with the Ki-67 index.

**Conclusions:**

This study suggests that the pons may be considered a suitable reference region for spinal ^18^F-FET PET imaging, which can improve the differentiation between spinal tumors and myelitis. The significant correlation between pons-based SUVR values and the Ki-67 index further highlights the potential of this approach in assessing tumor cell proliferation.

## Introduction

Positron emission tomography (PET) imaging has emerged as a valuable tool for assessing spinal diseases, offering functional insights into molecular processes occurring within the spinal cord [1]. By detecting metabolic activity and tracer uptake, which traditional imaging techniques, such as CT or MRI, could not illustrate, PET enables the characterization of various spinal conditions. Currently, PET has been utilized as a tool for the diagnosis, monitoring of treatment response, and even guiding surgical interventions for diseases such as tumors [2, 3], spondylosis [4, 5], amyotrophic lateral sclerosis [6–8], spinal cord injuries [9], multiple sclerosis [10] and so on.

Despite its utility, the interpretation of spinal PET results presents several challenges. A key challenge is the accurate selection of background reference regions, which affects the quantification and interpretation of the standardized uptake value ratio (SUVR) [11], a widely used quantitative metric in PET research. Unlike SUV, SUVR undergoes normalization based on a background region, which effectively reduces the impact of inter-individual physiological variations on statistical results. Many brain disease studies have demonstrated a close relationship between SUVR and clinical assessments [12–14]. However, inadequate consideration of background regions may lead to misinterpretation of findings and erroneous clinical decisions. Especially in spinal PET, the uptake signal is lower and more susceptible to the complex surrounding tissue compared to brain PET [1]. Therefore, the selection of an appropriate background region is crucial for spinal PET research.

Areas such as the whole brain [15, 16], cerebellum [17, 18], and pons [19, 20] are commonly used as background reference regions in brain PET studies. However, there is no consensus on the optimal choice of background reference regions for spinal PET analysis. Previous studies have suggested various anatomical structures, including supratentorial brain tissue [2], the aortic blood pool [21], the liver blood pool [22, 23], the spinal cord at C7/T1 [4], the distal spinal cord [24, 25], and different segments of the spinal cord [26], as potential background regions. However, the proposed regions vary significantly in their anatomical extent, spanning across several body parts. Furthermore, some studies, despite selecting a background region, relied solely on semi-quantitative methods such as subjective ratings [22, 27]. As a result, there has been a lack of comparative evaluations of the effects of different background region choices. The lack of standardized protocols for selecting background regions in spinal PET, due to discrepancies in study methodologies and limited comparative assessments, compromises the reproducibility and reliability of spinal PET findings across different clinical settings.

The primary objective of this study is to address the challenges associated with spinal PET analysis by evaluating and comparing different background reference regions. Using spinal tumors and myelitis as examples, we compared the stability of uptake in pons and some regions of the spinal cord (i.e., C2, C2–C7, and T1–T3) and the effect of SUVR values calculated with these areas as background to differentiate between the two diseases. In addition to this, we further compared the correlation between SUVR and tumor cell proliferation under different background selection. By systematically comparing the effectiveness of these reference regions, our study aims to identify the most appropriate reference region for accurate spinal PET analysis. Ultimately, this study aims to increase diagnostic accuracy, improve patient management strategies, and facilitate better clinical outcomes for individuals with spinal diseases.

## Materials and methods

### Patient population

The clinical study received approval from the ethics committee of our institution and obtained written informed consent from all participants. A total of 15 patients with cervical myelitis and 15 patients with cervical tumors were recruited from the hospital outpatient service. Diagnostic criteria for myelitis were referenced from prior literature [28]. Tumor patients were preliminarily diagnosed through a collaboration between spinal neurosurgeons and radiologists, with final diagnosis confirmed through pathological diagnosis. Besides, we evaluated tissue samples for the Ki-67 index, which is used to measure the proliferative activity of tumor cells [29]. The exclusion criteria included claustrophobia, metallic implants, cardiac pacemakers, permanent contraceptive devices, and other conditions incompatible with PET or MRI scanning.

### Imaging protocol

Before undergoing PET/MR examination, all subjects adhered to a fasting period of at least 12 h [30]. Subsequently, the subjects underwent scanning using the simultaneous PET/MR system (uPMR 790, United Imaging). The imaging protocol involved the injection of O-(2-[^18^F]-fluoroethyl)-L-tyrosine (FET), with the scan commencing approximately 60 min after uptake. The injected dose was 3 MBq of ^18^F-FET per kilogram of body weight [2]. Throughout the uptake period, patients remained in a dimly lit room without engaging in conversation. Following this, a cervical spine PET/MR scan was initiated simultaneously.

The subjects were positioned supine with arms down at their sides. To reduce the patient’s cervical curvature, we placed a soft cushion under the patient’s head before scanning. PET attenuation correction was performed using a T1-weighted 3D Dixon sequence MR scan [31]. PET data acquisition lasted for 20 min across 320 slices. Reconstruction of PET raw data on the console involved two iterations and 20 subsets, employing the ordered-subsets expectation–maximization algorithm incorporating both time-of-flight and point-spread function techniques. Additionally, PET images were reconstructed using a Gaussian filter with a full width at half maximum of 3.0 mm and resized into a 192 × 192 matrix. The voxel size was 3.13 × 3.13 × 2.80 mm^3^.

**Fig. 1:**
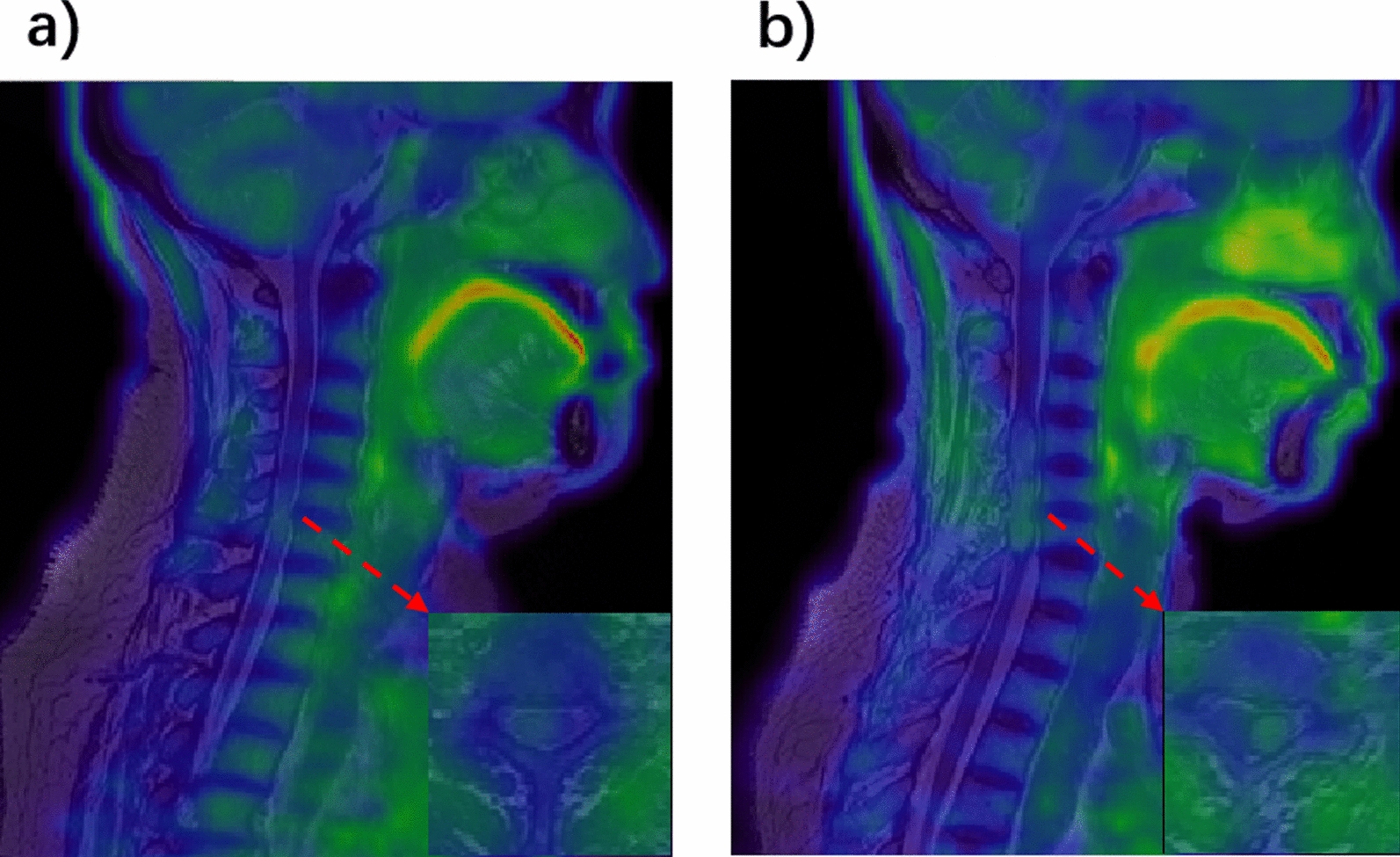
Examples of PET/MR fusion images. **a** Patients with myelitis (C5–6 affected). **b** Patients with spinal cord tumor (C4–6 affected). The lesions exhibit increased FET uptake

Additionally, a high-resolution T2-weighted images were acquired with a MATRIX sequence (TR/TE = 1500/121 ms, matrix size = 256 × 256, slice thickness = 0.8 mm, voxel dimensions = 0.8 × 0.8 × 0.8 mm^3^, sagittal acquisition). The MRI scan was centered at the C4/C5 intervertebral disc, covering from the Pons to the lower edge of the T3 vertebra. Additionally, standard diagnostic sequences commonly employed in clinical practice, including T1 and T2 weighted images, as well as diffusion-weighted images, were also acquired to precisely localize specific lesion areas. Figure 1 displays examples of fusion images derived from these two groups.

### Image analysis

PET and MRI data were processed using the FMRIB Software Library version 6.0.3 (FSL; http://www.fmrib.ox.ac.uk/fsl/) and Spinal Cord Toolbox version 4.0.0 (SCT; https://sourceforge.net/p/spinalcordtoolbox/wiki/tools/). Spinal cord segmentation masks were acquired from high-resolution T2-weighted images using SCT and manually corrected with FSL eyes if necessary. Following that, the structural image of each participant was registered to the PAM50 template using the SCT registration tools (sct_register_to_template and sct_warp_template) with forward and backward warping fields generated. Once registration was estimated, the regions of interest (ROI) in PAM50 template could warp back into native space.

**Fig. 2:**
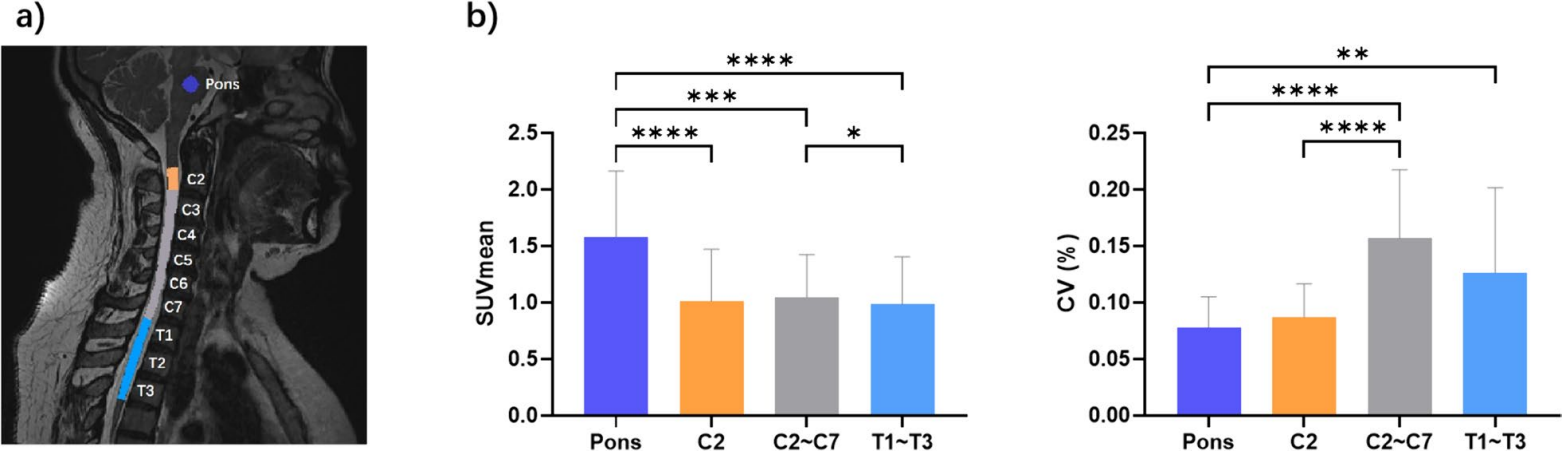
Comparison of SUVs in the affected areas between patients with myelitis and tumors. Regardless of SUVmax or SUVmean, there was no significant difference between the two groups

We defined four background regions: Pons, C2, C2–C7, and T1–T3. The cervical segments C2, C2–C7, and T1–T3 were automatically delineated using the PAM50 template registered into individual space (see Fig. 2a), and pons is drawn in the individual space. The Pons ROI was defined as follows: initially, a voxel mask was manually generated at the center of the Pons in each subject's high-resolution T2 image using FSLeyes. Subsequently, this voxel was transformed into a spherical ROI with a diameter of 9 mm using the fslmaths command (as referenced in [32]). Due to variations in voxel sizes and scan coverage, we utilized SCT software to register PET images with high-definition structural images of individuals before calculating the uptake index. Once the registration is complete, ROIs from individual structural images can be used directly for metrics extraction from PET images.

For uptake at the lesion location, professional imaging physicians examine segments of lesion areas for each patient using PET and T2-weighted images (example shown in Fig. 1). Then, for segments with lesions, the maximum uptake was measured as SUVmax. The SUVmean of the tumor area was calculated from the mean uptake within the tumor volume delineated using a threshold equivalent to 40% of SUVmax [33]. The SUVmean algorithm was the same for myelitis lesion areas.

The SUVmean and CV were calculated from these four background regions. The CV was defined as the Standard Deviation (SD) of SUV normalized to SUVmean in background region. Once SUVmean and SUVmax are obtained for each patient, different SUVRmean and SUVRmax values can be calculated from different background regions.

### Statistics

The study employed the Friedman test to compare SUVs and SUVRs obtained from various background regions. Post-hoc analysis was conducted using Dunn's test. Differences in uptake between the two groups of patients with myelitis and tumor were tested using Mann–Whitney test. Finally, Spearman's correlation analysis was used to correlate the SUVRmean and SUVRmax values of tumor areas obtained in different background regions with Ki-67. Statistical significance was determined by a p-value of less than 0.05.

## Results

### Demographic and clinical description

15 myelitis patients (mean age ± standard deviation (SD): 54.60 years ± 16.47) and 15 tumor patients (mean age ± SD: 50.00 years ± 13.87) were included in this study. There were no significant differences in age (*p* = 0.42) and sex (*p* = 0.71) in these two groups. Demographic details are presented in Table 1, with specific diagnoses, affected area locations, WHO grades, and Ki-67 scores for tumor patients detailed in Table 2.

**Table 1:** Summary of patient characteristics

Characteristics	Myelitis (*n* = 15)	Tumor (*n* = 15)	*p* value
Sex (M/F)	6/9	7/8	0.71^a^
Age (years)	54.60 ± 16.47	50.00 ± 13.87	0.42^b^
Height (cm)	164.00 ± 7.84	161.20 ± 20.56	0.63^b^
Weight (kg)	65.20 ± 13.60	64.13 ± 17.63	0.85^b^
Ki-67 (%)	–	15.93 ± 8.80	–

Data are represented as mean ± standard deviation

^a^Chi-squared test

^b^Two-sample t-test

**Table 2:** Detailed diagnostic information for patients with spinal cord tumors

Patient no	Sex	Age (yrs)	Diagnosis	Location	WHO grade	Ki-67 (%)
1	M	44	Glioma	C6-7	2	10
2	F	40	Ependymoma	C5-7	2	21
3	M	60	Ependymoma	C4-7	2	20
4	F	28	Ependymoma	C2-7	2	31
5	F	59	Ependymoma	C3-6	2	25
6	M	54	Ependymoma	C3-6	2	9
7	F	41	Glioma	C6-7	2	8
8	F	22	Ependymoma	C2-7	2	22
9	M	69	Ependymoma	C4-5	2	18
10	F	52	Ependymoma	C6-7	2	3
11	F	71	Ependymoma	C2-3	2	18
12	M	48	Ependymoma	C4-7	2	20
13	M	44	Ependymoma	C2	2	7
14	F	58	Ependymoma	C5-7	2	25
15	M	60	Ependymoma	C7	2	2

### FET uptake manifestations of myelitis and tumors in the lesion

**Fig. 3:**
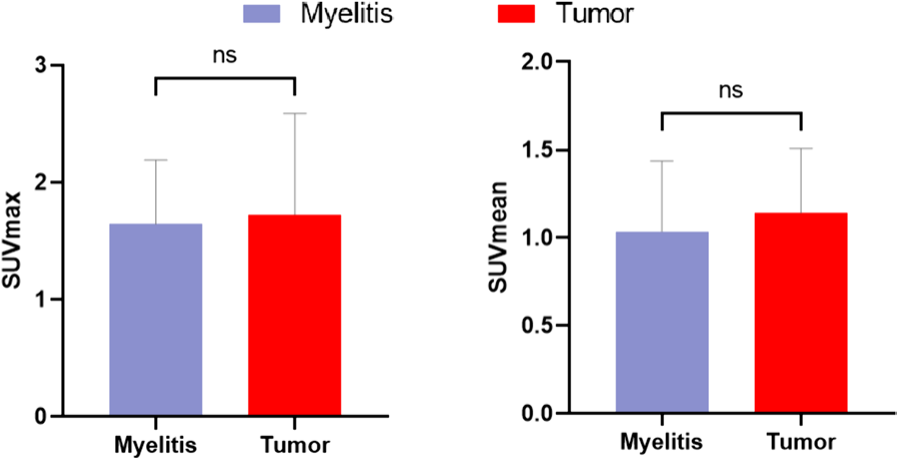
Comparison of uptake values from different background regions. **a** Illustration of background region selection. **b** Comparison of background metabolic stability. Mean uptake values were significantly higher in Pons than in other background regions (Left). Pons and C2 regions have lower coefficients of variation (Right)

Both myelitis and tumor patients showed increased uptake in the lesion locations (Fig. 1). However, the Mann–Whitney test did not reveal any significant differences in either SUVmax (U = 110, *p* = 0.93) or SUVmean (U = 89, *p* = 0.36) at the lesion sites between the two groups (see Fig. 3).

### Stability of uptake in different background regions

Significant differences in mean uptake value were observed among various background regions (Fr = 58.48, *p* < 0.0001, Fig. 2b left). Post-hoc comparisons revealed that the Pons had significantly higher uptake value compared to C2 (Z = 6.40, *p* < 0.0001), C2–C7 segments (Z = 4.00, *p* = 0.004), and T1–T3 segments (Z = 6.80, *p* < 0.0001). Additionally, the mean uptake in C2–C7 segments was significantly higher than in the upper thoracic segments (Z = 2.80, *p* = 0.03). Significant differences in uptake variability were observed across different reference regions (Fr = 42.68, *p* < 0.0001, Fig. 2b right). The coefficient of variation (CV) was significantly lower in the Pons compared to C2–C7 (Z = 6.10, *p* < 0.0001) and T1–T3 (Z = 3.90, *p* = 0.006). Additionally, the variability in the C2 region was also significantly lower than that of C2–C7 segments (Z = 4.50, *p* < 0.0001). However, there was no significant difference in CV between the Pons and C2 (Z = 1.60, *p* = 0.67).

### Differentiation of SUVRs obtained from various background regions between the two diseases

**Fig. 4:**
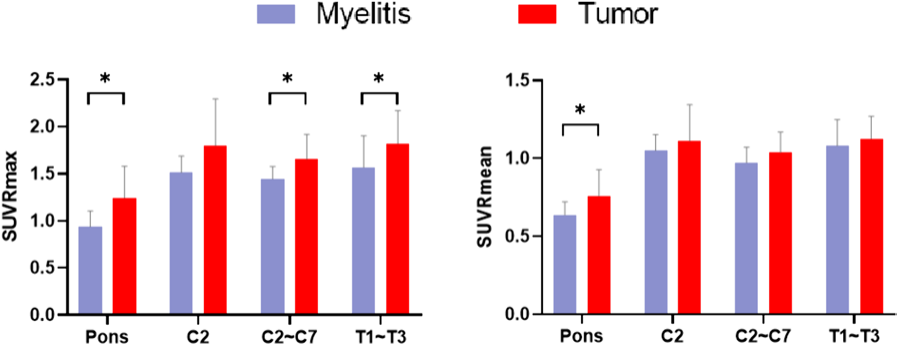
Comparison of SUVRs between tumors and myelitis in various background regions. SUVRmax obtained from Pons, C2–C7, and T1–T3 were all able to distinguish uptake differences in tumor and myelitis (Left). The SUVmean obtained from the Pons was the only effective measure in distinguishing uptake between tumors and myelitis (Right)

The distinction between tumors and myelitis is significantly affected by the differentiation of SUVRs by background regions (Fig. 4). SUVRmax of tumor obtained with Pons is significantly higher than that of myelitis (U = 52, *p* = 0.01, Fig. 4 left). Similarly, tumor SUVRmax obtained with C2–C7 (U = 62, *p* = 0.04) and T1–T3 (U = 63, *p* = 0.04) as backgrounds are also significantly higher than that of myelitis. However, only the SUVRmean derived from Pons can significantly distinguish between these two groups (refer to Fig. 4 right). The tumor SUVRmean is significantly higher than that of myelitis (U = 62, *p* = 0.04). SUVRmean obtained from other regions does not effectively differentiate uptake differences between the two groups.

### The correlation between SUVRs obtained from different background regions and Ki-67 in tumor patients

**Fig. 5:**
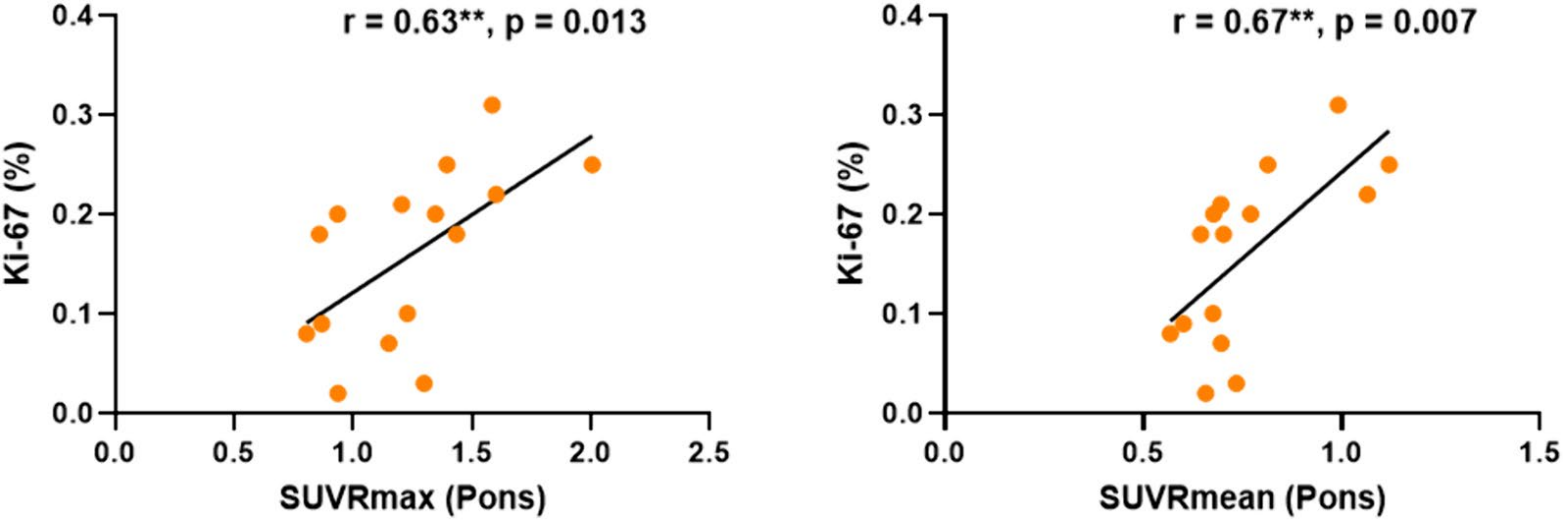
SUVRmax and SUVRmean obtained with Pons as background were significantly positively correlated with Ki-67 values

Only SUVRmax (*r* = 0.63, *p* = 0.013) and SUVRmean (*r* = 0.67, *p* = 0.007) obtained from the Pons background showed a significant positive correlation with Ki-67 (refer to Fig. 5). There was no significant association between SUVRs obtained from other regions and Ki-67, as detailed in Table 3.

**Table 3:** Correlation of SUVRs calculated from different background regions with Ki-67

Background region	Pons	C2	C2–C7	T1–T3
SUVRmax				
r	0.64	0.14	0.08	0.45
p	< 0.01	0.62	0.78	0.09
SUVRmean				
r	0.67	0.20	− 0.06	0.46
p	< 0.01	0.49	0.84	0.09

## Discussion

This study presents a comprehensive evaluation of various background reference regions for spinal PET analysis, with a specific focus on spinal cord tumors and myelitis. It is one of the first systematic attempts to identify the most effective reference region for improving diagnostic accuracy and differentiating between these two diseases using PET imaging. Our findings indicate that there were no significant differences in SUVmax and SUVmean values at the lesion sites between myelitis and tumor patients. However, we did observe significant variability in uptake values across different proposed background regions. Specifically, the pons region demonstrated higher stability and lower variability in uptake compared to cervical and thoracic spinal cord segments. The study's findings suggest that SUVR values, specifically those obtained from the pons, can effectively distinguish between spinal tumors and myelitis. Additionally, a significant positive correlation was found between SUVRmax / SUVRmean from the pons and the Ki-67 proliferation index in tumor patients, highlighting the potential of pons-based SUVR measurements in reflecting tumor cell proliferation activity.

Our study found no significant difference in FET uptake, as measured by SUV, between spinal cord tumors and myelitis. This finding contradicts previous studies, which often associated increased FET uptake with tumor presence. PET imaging with FET has historically been useful in identifying tumors due to the tracer's tendency to accumulate in regions of high amino acid transport, a hallmark of tumor cells [34, 35]. A wealth of research has already demonstrated the effectiveness of using FET in distinguishing between tumorous and non-tumorous diseases [36]. These findings may support the expectation of higher FET uptake in tumors compared to inflammatory conditions due to the metabolic demands of proliferating tumor cells, and this has been validated in animal studies [37].

There may be several reasons for the absence of significant differentiation between the SUV of the tumor and myelitis. First, the small size of the spinal cord and its proximity to other structures may complicate the accurate assessment of uptake [1], leading to potential overlap in SUV values. Second, the inflammatory response in myelitis may involve increased amino acid transport and metabolism, as reported by Hayashi et al. [38], mimicking the metabolic patterns seen in tumors. Additionally, it is important to note that PET manifestations can vary depending on the type of tumor and stage of inflammation [39, 40]. Furthermore, technical aspects of PET imaging, such as resolution, time of tracer uptake measurements, and patient movement, may also contribute to the lack of distinct SUV values between these conditions. This variability may further complicate the distinctions typically observed through PET imaging. It should be noted that the degree of myelitis in each patient with inflammation was not assessed in this study, while the WHO grades in patients with tumors were predominantly mild grade II. These factors may contribute to the obscuring of direct uptake differences between tumors and inflammation.

Our findings suggest that SUVs in myelitis may be comparable or slightly higher than in spinal tumors, although the difference is not statistically significant. This could be due to the intense inflammatory response associated with myelitis, which may involve active cellular proliferation, increased metabolic activity, and enhanced amino acid transport [38], similar to neoplastic processes. Inflamed tissues may contain activated macrophages and lymphocytes [41], which could exhibit increased metabolic activity. Our findings suggest that using SUV values alone to distinguish between spinal pathologies may be limited. The overlap in SUV between tumors and myelitis indicates that this metric alone may not provide sufficient specificity for accurate disease characterization in the spinal cord. On the other hand, normalizing SUV values to generate SUVR, by selecting an appropriate and stable background reference region, appears to offer greater specificity.

The stability of the uptake in the background region is critical for calculating SUVRs. SUVR normalizes the uptake in the target region against the reference region in the background to mitigate individual physiological variations. Therefore, the selection of the background reference region could affect the accuracy and reliability of PET analysis. Our study revealed a distinct uptake pattern among the evaluated regions. The pons exhibited higher uptake values compared to the other three regions, which may be attributed to its unique functional role. Previous studies have shown that different brain regions and other parts of the body exhibit varying degrees of uptake, even in the absence of lesions [42]. This may be due to the metabolic demands and functional activities specific to each region. For example, the researchers observed that explicit neural activity would require more glucose uptake compared to oxygen consumption alone. Additionally, different regions of the brain exhibit differences in metabolic uptake [43]. The pons, a crucial part of the brainstem, plays a pivotal role in regulating vital functions such as respiration, sleep, and arousal [44]. This may contribute to its higher metabolic activity and, consequently, higher PET uptake. Considering its role as a crucial conduit for sensory input and motor output signals [45], the spinal cord may exhibit relatively lower metabolic activity and PET uptake compared to the pons. This difference highlights the more straightforward functional role of the spinal cord, in contrast to the pons' complex involvement in regulating vital functions, which may contribute to its higher baseline metabolic activity and PET uptake.

Although the pons showed a higher uptake, it had the lowest CV. The CV measures uptake variability relative to the mean value, providing a measure of metabolic consistency or fluctuation. Factors that can influence metabolic variability range from physiological variations [46], such as cyclic or situational changes in brain activity, to technical aspects of PET imaging [47]. The pons is characterized by low variability, which reflects its metabolic stability and is consistent with its constant and crucial functional demands. It plays a vital role in various autonomic functions, such as regulating respiration, heart rate, and sleep cycles [44]. Additionally, the pons contributes to the coordination of movement and the processing of sensory information, which may also contribute to its metabolic stability. The pons plays critical roles, and therefore, its metabolic demands are high and need to be consistently maintained to support its continuous and complex functional activities. Compared to other regions, the pons experiences lower metabolic fluctuation, making it an appropriate reference region. This ensures more reliable normalization of target region uptakes and enhances diagnostic accuracy in spinal PET studies.

Although there were no significant differences in SUV values between spinal tumors and myelitis, normalization through SUVR calculation revealed a consistent elevation in tumors across all considered background regions. This observation suggests that SUVRs, by accounting for inter-individual uptake variability, might offer a more consistent marker for disease characterization in spinal PET imaging than SUVs alone. SUVRs have been found to provide a clearer distinction between disease states and healthy controls or between different pathological conditions in brain tumor and neurodegenerative disease studies [48, 49]. This highlights the potential of SUVR as a more robust tool for disease differentiation across varied clinical contexts. It is important to note that the choice of background reference significantly influences the discriminatory power of SUVRs. The study indicates that SUVRmax values obtained using the pons, entire cervical spinal cord, and upper thoracic spinal cord as reference regions can effectively differentiate between tumors and myelitis. However, only SUVRmean values calculated with the pons as the background provide a reliable differentiation. The specificity of the pons may be attributed to its metabolic stability, as previously discussed. This stability ensures a consistent baseline for normalization, enhancing the reliability of SUVR in distinguishing between pathological conditions.

The impact of selecting a background reference region on diagnosis has been observed in other studies. For instance, Li et al. conducted a study on neurodegenerative diseases and found that the calculated SUVR was more effective in distinguishing between cognitively normal and cognitively impaired Alzheimer's patients when using the pons as the reference region compared to cerebellum gray matter and centrum semiovale [50]. In our study, the pons demonstrated superior performance in differentiating tumors from myelitis, both for SUVRmean and SUVRmax values, corresponding to its stable uptake characteristics. This reaffirms its suitability as a background reference in spinal PET analysis. However, it is important to note that the common diagnostic thresholds, such as SUVRmax of 2.1 and SUVRmean of 1.6, which are used to distinguish between primary neoplastic lesions and non-neoplastic lesions [51], were not applicable in our spinal study. All calculated SUVR values fell below this benchmark. This highlights the importance of having specific standards for selecting background regions and SUVR thresholding in the spinal cord, considering its distinct metabolic and functional characteristics compared to the brain.

Therefore, we further explored the correlation of SUVR values derived from various background regions with clinical assessments. To ensure the clinical utility of any biomarker, criterion validity is crucial, which requires testing its measurement against true clinical outcomes or established clinical indices. Previous studies have demonstrated significant correlations between SUVR values and various clinical indices for different brain disorders [52, 53]. For instance, Ottoy et al. discovered that SUVR values calculated with white matter were significantly correlated with volume of distribution, emphasizing the clinical relevance of SUVR values in Alzheimer's Disease [52]. These findings emphasize the need to evaluate SUVR values against clinical outcomes to establish their validity and usefulness as effective biomarkers in clinical settings.

The Ki-67 index, a marker of cellular proliferation, was chosen to assess the criterion validity of SUVR values obtained from four different background reference regions. Ki-67 is an important clinical assessment tool in oncology as it indicates the growth rate of tumor cells [54]. Its correlation with FET uptake suggests that higher metabolic activity, as measured by PET, is associated with increased proliferation rates in tumors. This relationship highlights the potential utility of FET PET imaging for assessing tumor aggressiveness and could inform treatment strategies. Our analysis revealed that only the SUVR values calculated with the pons as the background exhibited a significant correlation with the Ki-67 index (Fig. 5, Table 3). The correlation between SUVR values derived from the pons and Ki-67 validates the clinical relevance of this approach. This provides a strong foundation for its use in improving diagnostic precision and patient management in the context of spinal disorders.

The study has limitations due to a small sample size and a focus primarily on WHO grade II ependymomas, which may limit the generalizability of the results. To address these limitations, future research should expand the participant pool to include a larger and more diverse group, encompassing a variety of spinal tumor types and grades. Furthermore, it is important to note that this study exclusively utilized FET as a tracer. Future studies may benefit from exploring the rationality of pons as a background reference region for spinal PET by utilizing other tracers. The reference region selected in this study is concentrated around the neck due to the influence of the visual field and acquisition range. However, it may be beneficial to acquire PET images of the brain or larger regions in the future to test the possibility of utilizing additional regions as background references.

## Conclusions

SUVR obtained with pons as background region can effectively distinguish myelitis from spinal cord tumors and is closely correlated with the tumor invasion index. Pons is a suitable background reference region for PET assessment of the spinal cord.

## Data Availability

The datasets used and analyzed during the current study are available from the corresponding author on reasonable request.

## Abbreviations

FET: O-(2-[^18^F]-fluoroethyl)-L-tyrosine
PET: Positron emission tomography
SD: Standard deviation
SUVR: Standardized uptake value ratio
